# Case Report: Virtual and Interactive 3D Vascular Reconstruction Before Planned Pancreatic Head Resection and Complex Vascular Anatomy: A Bench-To-Bedside Transfer of New Visualization Techniques in Pancreatic Surgery

**DOI:** 10.3389/fsurg.2020.00038

**Published:** 2020-06-18

**Authors:** Robert Templin, Navid Tabriz, Martin Hoffmann, Verena Nicole Uslar, Thomas Lück, Andrea Schenk, Rainer Malaka, Gabriel Zachmann, Alexander Kluge, Dirk Weyhe

**Affiliations:** ^1^University Hospital for Visceral Surgery, Pius-Hospital, Carl von Ossietzky University Oldenburg, Oldenburg, Germany; ^2^cirp GmbH, Heimsheim, Germany; ^3^Fraunhofer Institute for Digital Medicine MEVIS, Bremen, Germany; ^4^Digital Media Lab, University of Bremen, Bremen, Germany; ^5^Computer Graphics and Virtual Reality, University of Bremen, Bremen, Germany; ^6^Department of Diagnostic and Interventional Radiology, Pius-Hospital, Oldenburg, Germany

**Keywords:** Bühler anastomosis, digitization, virtual reality, augmented reality, pancreas, 3D-printing

## Abstract

**Introduction:** Bühler's anastomosis (or Bühler's arcade) is an embryonic relic and represents an arterio-arterial connection between the superior mesenteric artery and the celiac trunk. It can be found as a variety in 1–2% of patients.

**Case Presentation:** We present a case of a patient with metatastatic squamous cell carcinoma of the lung. The patient was in stable disease for 4 years under palliative therapy (most recently second-line therapy with Nevolumab). In 2019, a locally advanced adenocarcinoma of the papilla vateri was diagnosed, additionally. The patient also underwent right hemicolectomy and patch plasty of the celiac trunk and superior mesenteric artery due to colonic ischemia and arteriosclerotic disease with 50–70% stenosis of the superior mesenteric artery several years ago. Due to a complex vascular prehistory, the standardized preoperative imaging was supplemented by two independent vascular reconstructions (a CT angiogram and a reconstruction based on the CT) for the planning of a pylorus-preserving pancreatic head resection and reconstruction according to Traverso-Longmire. In addition, a 3D print was produced. Both, the reconstruction based on the CT scan and the 3D print were created for off-label use as a part of a research project (VIVATOP: Versatile Immersive Virtual and Augmented Tangible OP).

**Discussion:** In the standardized CT scan and in the clinical CT-angiography, there were no obvious surgically relevant anatomical variations. A Bühler anastomosis was detected in a digital, virtual and interactive 3D-reconstruction. In addition, in the 3D print of the abdominal site the anastomosis was seen as well. Intraoperatively, the presence of Bühler's anastomosis was confirmed. This information had a significant impact on the intraoperative approach. Retrospectively, the vessel variant could be surmised in the axial projection of the CT scan, if one knew what to look for.

**Conclusion:** For the conduction of a safe surgical procedure, it is imperative that rare anatomical variations are known preoperatively. Increasing digitalization in surgical and perioperative preparation holds great potential for better planning and improved patient safety. Research and cooperation projects such as the VIVATOP project are instrumental for the development of new visualization techniques, which are able to enhance the understanding of complex anatomical relations.

## Introduction

The Bühler anastomosis (also Bühler arcade) was first described in 1904 (1). It represents an arterio-arterial connection between the superior mesenteric artery and the celiac trunk. It is thought to occur as an embryonic relic as a vessel variant in 1–2% of patients (2).

Hereby, we present the case of a patient with known metastatic squamous cell carcinoma of the lung which has been in stable disease for 4 years under a second-line palliative therapy with Nevolumab and recently diagnosed locally advanced adenocarcinoma in the pancreatic head region, most likely of the papilla vateri. Based on a complex history of abdominal vascular disease, we decided to expand the preoperative imaging via CT-angiography, an additional digital 3D vascular reconstruction and a 3D print of the abdominal site as part of a research project.

## Case Presentation

**Figure 1:**
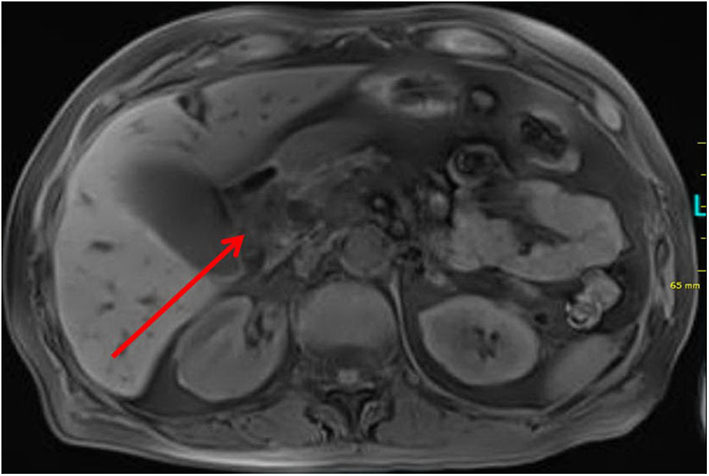
MRI scan showing the tumor in pancreatic region in axial projection.

A 71-year-old patient admitted to an outside hospital due to fatigue and increased jaundice. Standard blood exams revealed pathologic findings of cholestasis parameters (bilirubin, 5.8 mg/dL, direct bilirubin, 5.3 mg/dL, GGT, 1262 U/L, AP, 941 U/L), transaminase (GPT, 472 U/L), and lipase (227 U/L). A MRCP and CT scan displayed intra- and extrahepatic cholestasis as well as a tumor in the pancreatic head region (Figure 1). The patient was referred to our department for planning further treatment. An endoscopic retrograde cholangiopancreaticography with consecutive stenting of the common bile duct was performed. Endoscopic ultrasound confirmed the tumor in the pancreas head region shown in the aforementioned scan. The tumor was punctured. The histopathological work-up yielded the finding of an adenocarcinoma. At this time, it could not be differentiated with certainty between a papillary or pancreatic carcinoma.

Due to a previously known metastatic lung carcinoma, which has been in stable disease under second-line palliative therapy with Nevolumab for years, we first presented the case in our interdisciplinary tumor conference. Prognosis of the disease was difficult. Overall, it was expected that the patient might survive further years under palliative therapy.

Additionally, the patient suffered from a primary adrenal insufficiency with permanent cortisone medication. Furthermore, the patient had to undergo right hemicolectomy and patch plastic of the celiac trunk and superior mesenteric artery due to colonic ischemia and arteriosclerotic disease with 50–70% stenosis of the superior mesenteric artery several years ago (alio loco).

After a detailed discussion with the patient and in particular explaining the possible risks and complications of a pancreatic head resection with consecutive reconstruction, as well as existing consensus of all in the treatment involved disciplines, the operation was performed. For the planning of a pylorus-preserving pancreatic head resection and reconstruction according to Traverso–Longmire, the preoperative imaging was extended by two independent vascular reconstructions. At this point informed consent was acquired from the patient for the use of data gathered during his stay in a potential publication. Since treatment did not deviate from normal treatment in those cases, no ethical approval was needed, according to the guidelines of the medical ethics committee of the Carl von Ossietzky University Oldenburg.

**Figure 2:**
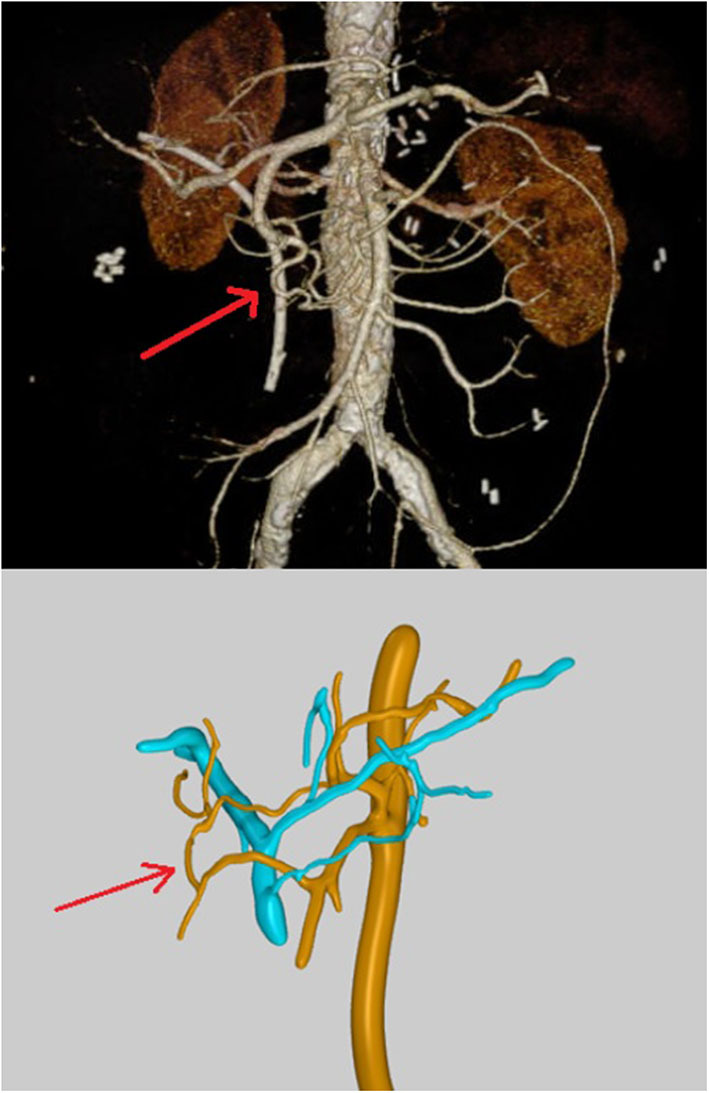
Upper panel: CT-angiography showing only a prominent Riolan anastomosis and possibly partly re-stenotic patch plastics of the celiac trunk and superior mesenteric artery; the red arrow points to a vessel that appears like a terminal branch of the gastroduodenal artery without any connection to the superior mesenteric artery; lower panel: virtual 3D-vascular and interactive reconstructions showing the Bühler anastomosis as connection between the superior mesenteric artery and the celiac trunk.

**Figure 3:**
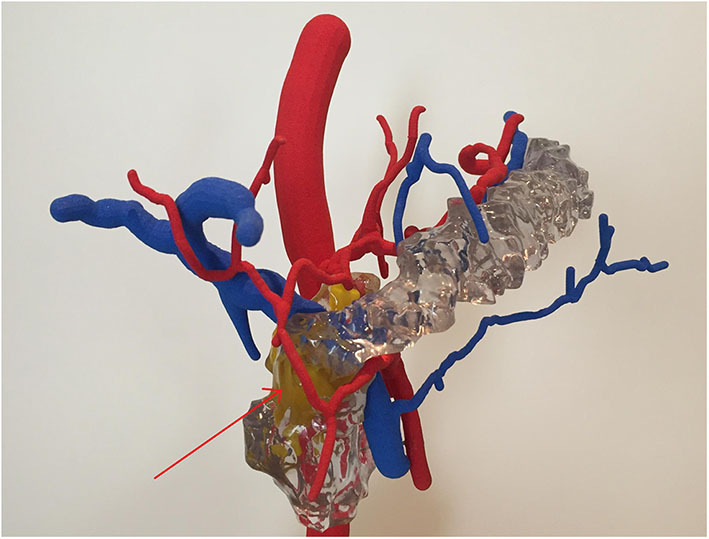
3D print of the abdominal site clearly showing the Bühler anastomosis; the yellow structure in the background represents the tumor. Because of the lack of close relationship to the vessel, an oncological resection with preservation of the vascular connection was technically feasible.

While the actual clinical CT-angiography only showed a prominent Riolan anastomosis and possibly partly re-stenotic patch plastics of the celiac trunk and superior mesenteric artery, the Bühler anastomosis was only detected in the digital 3D reconstruction, and it was conspicuous in the 3D print, which both have been created with the consent of the patient as an off-label use in the research project VIVATOP. Figure 2 shows the results of the two different visualization techniques. With the visualization depicted in Figure 2 (lower panel), a 360° representation is also possible. In addition, the virtual viewing in VR glasses is feasible. Figure 3 shows the 3D print derived from the reconstruction depicted in Figure 2 (lower panel) with the pancreas added.

Intraoperatively, the presence of Bühler's anastomosis was confirmed. However, this information had a significant impact on the intraoperative approach. Due to a possibly inadequate blood supply via the partly stenotic patch plastics of the celiac trunk and superior mesenteric artery the vessel connection could not be removed in the course of the pancreatic head resection. If the surgeons performing the surgery would not have reacted with the additional information in mind and cut the anastomosis, the patient would have been at a high risk for mesenteric ischemia. The oncological resection was nevertheless technically feasible.

The intra- and postoperative course was accompanied by mild cholangitis without further complications. The definitive histopathological findings finally revealed a locally advanced adenocarcinoma of the papilla vateri [pT3b pN2 (7/13) cM0 L1 V1 pn1 R0—according to UICC-stage IIIb], which has a much better prognosis than pancreatic carcinoma even in cases of lymph node involvement (3).

**Figure 4:**
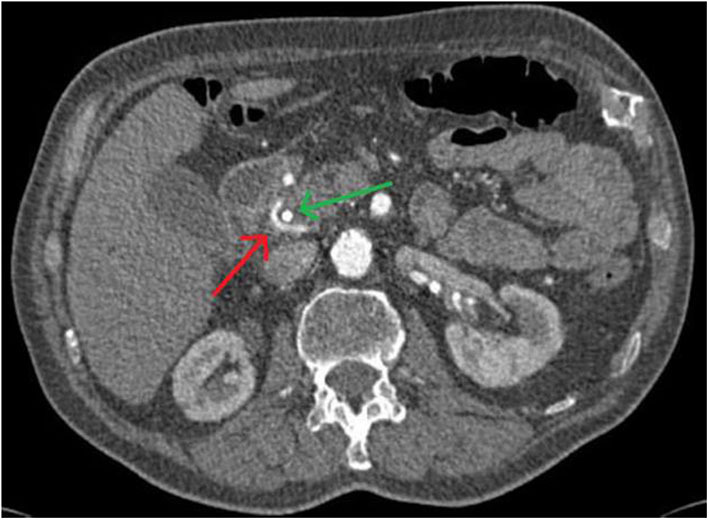
The Bühler anastomosis can be surmised in the axial projection of the CT scan. The red arrow points to the vascular variant, the green one to the stent in the hepatic duct.

Retrospectively, this vessel variant could be surmised in the axial projection of certain slices of the CT scan by the radiologist, when they knew what to look for (Figure 4).

## Discussion

Anatomical variations in the area of the abdominal vessels occur much more frequently than expected. Some authors report up to 39% of patients deviating from anatomy as described in anatomical textbooks (4, 5). Most of those variations are only minor and have no impact on the respective person, even during surgery. However, in the case described here, the additional information gained from the 3D reconstructions had significant impact on the intraoperative strategy.

In the age of digitization, one of the main goals of the VIVATOP project is the implementation of virtual and augmented reality into everyday clinical practice. This includes both the preoperative planning, as well as the intraoperative support and not at least training and education (6). First positive experiences have already been made in connection with this project, especially in the field of 3D printing (7).

Clinical studies about the benefits of those new visualization techniques and 3D prints are still very rare worldwide, but especially in Germany. A research team consisting of members of the Fraunhofer Institute for Medical Image Computing (MEVIS, Bremen, Germany) and Friedrich Alexander University (Erlangen-Nürnberg, Germany) has already shown that complex medical information can be presented easily and platform-independent. Both medical staff and patients could benefit from the possibilities of a 3D presentation (8).

Sheik-Ali et al. (9) recently conducted a literature review of recent comparative studies related to the impact of Phase 2 VR or AR tools on surgical training. Eleven studies on the effectiveness of VR/AR in surgical education have been reviewed. All studies showed a positive association between the use of VR/AR in surgical training and skill acquisition (improving the speed of acquisition of surgical skills, the surgeon's ability to multitask, the ability to perform a procedure accurately, hand-eye coordination and bimanual operation).

A similar assessment was made by Lesch et al. (10) in a study of 37 medical students regarding initial steps in laparoscopic surgery. In the VR group, there was a greater confidence in the ability to reproduce procedural steps (*p* < 0.001). Wada et al. (11) came as well to the conclusion that virtual 3D simulations are a useful educational tool. This was studied in conjunction with a laparoscopic TAPP on 30 medical students. In a meta-analysis, Guedes et al. (12) came to the conclusion that the VR-based training is superior to those traditionally used by box-trainers with regard to some interventions.

A Dutch research group led by Kubben et al. (13) has already shown that a head-mounted augmented reality device can be used routinely and sterile in the operating room even under different lighting conditions and using different surgical gloves. However, this is one of only a few studies that tried to implement new visualization techniques in the operating theater. Thus, further exploration of this field seems highly relevant.

The limitation of the described approach is that currently not every center has the luxury of having the equipment or knowhow necessary to perform this work up before surgery. However, at least in Germany, there is a strong trend toward centralization for complex surgery of any kind. An approach like the one described here might therefore become an integral part of the perioperative work up in those centers, especially since, as is the case with all new techniques, the required equipment will certainly become cheaper in the foreseeable future.

## Conclusions

The present case not only impressively shows that interdisciplinary oncological therapy concepts often require a tailored approach, but also that the implementation of virtual and augmented reality and 3D printing are new promising tools for perioperative visualization. They have the potential to be instrumental in promoting patient safety.

## Data Availability Statement

The datasets generated for this study are available on request to the corresponding author.

## Ethics Statement

Ethical review and approval was not required for the study on human participants in accordance with the local legislation and institutional requirements. The patients/participants provided their written informed consent to participate in this study.

## Author Contributions

TL, RM, AS, VU, DW, and GZ contributed to the conception and design of the study. AS and AK did the CT analyses which provided the 3D visualizations. TL provided the 3D print. RT, NT, and MH performed the surgery described in this case report. RT wrote the first draft of the manuscript and prepared the pictures together with VU. All authors contributed to manuscript revision, read and approved the submitted version.

## Conflict of Interest

The authors declare that the research was conducted in the absence of any commercial or financial relationships that could be construed as a potential conflict of interest.
